# Long non-coding RNA ABHD11-AS1 promotes colorectal cancer progression and invasion through targeting the integrin subunit alpha 5/focal adhesion kinase/phosphoinositide 3 kinase/Akt signaling pathway

**DOI:** 10.18632/aging.203342

**Published:** 2021-08-10

**Authors:** Jia Luo, Yigui Jiang, Lianhui Wu, Dexiang Zhuo, Shengjun Zhang, Xiang Jiang, Yingming Sun, Yue Huang

**Affiliations:** 1 Department of Gastroenterology, Affiliated Sanming First Hospital of Fujian Medical University, Sanming 365000, Fujian, China; 2 Department of Endoscope Room, Affiliated Sanming First Hospital of Fujian Medical University, Sanming 365000, Fujian, China; 3 Department of Clinical Laboratory, Affiliated Sanming First Hospital of Fujian Medical University, Sanming 365000, Fujian, China; 4 Department of Gynecology, Affiliated Sanming First Hospital of Fujian Medical University, Sanming 365000, Fujian, China; 5 Department of Medical and Radiation Oncology, Affiliated Sanming First Hospital of Fujian Medical University, Sanming 365000, Fujian, China

**Keywords:** colorectal cancer, lncRNA-ABHD11-AS1, PI3K/Akt pathway, oncogene profile

## Abstract

Long non-coding (lnc)RNA ABHD11-AS1 participates in the development and progress of various cancers, but its role in colorectal cancer (CRC) remains poorly known. In the present study, public database analysis and quantitative reverse transcription PCR of CRC and normal tissues showed that ABHD11-AS1 was overexpressed in CRC and associated with poor prognosis in CRC patients. Both *in vitro* and *in vivo* experiments demonstrated that loss-of-function of ABHD11-AS1 attenuated the proliferation, migration, and invasion of CRC cells and induced their apoptosis. Transcriptome sequencing and Kyoto Encyclopedia of Genes and Genomes pathway enrichment analysis indicated that the phosphoinositide 3 kinase (PI3K)/Akt signaling pathway is a potential target of ABHD11-AS1. Additionally, we noted that ABHD11-AS1 deficiency reduced integrin subunit alpha (ITGA)5 expression, and impaired the phosphorylation of P85, focal adhesion kinase (FAK), and Akt1 in CRC cell lines and tumor tissues of nude mice. Furthermore, we observed that ITGA5 overexpression abrogated the effect of ABHD11-AS1 knockdown on the proliferation and invasion abilities of CRC cells. Taken together, our studies suggest that lncRNA ABHD11-AS1 promotes proliferation, migration, and invasion in CRC by activating the ITGA5/Fak/PI3K/Akt signaling pathway, and that the ITGA5/Fak/PI3K/Akt axis is a promising target for CRC therapy.

## INTRODUCTION

Colorectal cancer (CRC) has the third highest incidence of malignant tumors and the fifth major cause of mortality in China [1]. Although great progress has been made in its screening and treatment during the past decade, the prognosis for CRC patients is still poor and the 5-year overall survival rate is only 56.7% [2]. Moreover, the limitations of screening strategies mean that patients are typically diagnosed with advanced disease, which is associated with an unfavorable clinical outcome. Therefore, it is of great importance to identify a reliable and valid biomarker and molecular target for colorectal cancer.

LncRNAs exceed 200 nucleotides in length and lack a protein coding ability. They are important regulators of biological processes, including chromosome structure modulation, epigenetic regulation, transcription, mRNA splicing, and translation [3, 4]. LncRNAs such as RP11-317J10.2 [5], HOTAIR [6], CCAT2 [7], BCAR4 [8], and H19 [9] both activate and inhibit gene expression via a diverse range of mechanisms to affect CRC progression. However, to date, only a few lncRNAs associated with CRC have been characterized in detail.

LncRNA ABHD11 antisense RNA 1 (ABHD11-AS1), located in the 7q11.23 region, has a carcinogenic effect in multiple tumors. Wen et al. confirmed that ABHD11-AS1 enhanced cell multiplication and metastasis in papillary thyroid carcinoma and predicted poor survival [10]. Similarly, ABHD11-AS1 promoted proliferation, invasion, and migration, and inhibited the apoptosis of ovarian cancer cell lines [11]. Moreover, its tumorigenic potential was identified in endometrial carcinoma [12] and pancreatic cancer [13], where it was found to be associated with tumor–node–metastasis staging and prognosis.

The molecular mechanism by which ABHD11-AS1 affects the occurrence and development of CRC has not been fully investigated. In this study, we show that ABHD11-AS1 may function as an oncogene and that high ABHD11-AS1 expression is significantly correlated with poor prognosis in CRC patients. Additionally, lentivirus-mediated downregulation of ABHD11-AS1 was found to inhibit CRC cell proliferation, invasion and migration by down-regulating the integrin subunit alpha (ITGA)5/focal adhesion kinase (Fak)/phosphoinositide 3 kinase (PI3K)/Akt pathway, both *in vivo* and *in vitro*. Our results suggest that ABHD11-AS1 is a promising therapeutic target could be used to assess prognosis in CRC.

## MATERIALS AND METHODS

### Patient tissue samples

CRC tissues and paired normal tissues were collected from a total of 60 CRC cases admitted to the Sanming First Hospital Affiliated to Fujian Medical University between April 2013 and October 2014. All samples were independently confirmed to be adenocarcinoma by two pathologists. Samples were frozen in liquid nitrogen immediately after surgery until required for analysis. Patients who had received any neoadjuvant therapies or who had a history of cancer were excluded from the study. The Ethics Committees of Affiliated Sanming First Hospital of Fujian Medical University approved the study (approval no. 2013[72]), and all patients signed an informed consent form.

### Cell culture

The human colorectal cancer cell lines HT29, HCT116, SW480, SW620, and LoVo, and normal colonic epithelium cell lines NCM460 and FHC were cultured in DMEM medium with 10% fetal bovine serum (FBS), 100 units/ml penicillin, and 100 μg/ml streptomycin at 37° C with 5% CO_2_ [14].

### Plasmid construction

shRNA1(sh1) and shRNA2(sh2) specific for ABHD11-AS1 were synthesized and subcloned into PLKO.1-TRC-puro vector. ABHD11-AS1 sh1 sequences were as follows: forward, 5-CCGGGGACCAAGTCCTCCAGGAACGCTCGAGCGTTCCTGGAGGACTTGGTCCTTTTT-3, and reverse, 5-AATTAAAAAGGACCAAGTCCTCCAGGAACGCTCGAGCGTTCCTGGAGGACTTGGTCC-3; ABHD11-AS1 sh2 sequences were: forward, 5-CCGGTTCTCCGAACGTGTCACGTTTCAAGAGAACGTGACACGTTCGGAGAATTTTT-3, and reverse, 5-AATTAAAAATTCTCCGAACGTGTCACGTTCTCTTGAAACGTGACACGTTCGGAGAA-3. The coding region of ITGA5 was obtained by PCR method and was subcloned into pZsG vector. Primers for ITGA5 were: forward, 5' -ATAAGAATGCGGCCGCAAGAGCGGGCGCTATGGGG-3' and reverse, 5' -GACCTACGTAAATGGGAGTCTGAAATTGGGAGGACTC-3'.

### RNA extraction and quantitative reverse transcription PCR (RT-qPCR) assays

Total RNA was extracted using TRIzol reagent (Invitrogen, Carlsbad, CA, USA) and a total of 2 μg RNA was used to synthesize first-strand cDNA using a reverse transcription kit (Takara, Dalian, China). 18S was used as an internal control.

PCR primers were: ABHD11-AS1 forward, 5'-CTCTCCACCTGACAGCAACATC-3' and reverse, 5'- TTGGTCCAGGGAGGGTTCT-3'; and 18S forward, 5'- CGACGACCCATTCGAACGTCT-3' and reverse, 5'- CTCTCCGGAATCGAACCCTGA-3'. Relative transcript levels of ABHD11-AS1 were calculated using the 2^-ΔΔCt^ method. All experiments were performed in triplicate.

### Cell proliferation assay

HCT116 and SW480 cells were plated in 96-well plates at 3×10^3^ cells well and maintained in DMEM containing 10% FBS. At each indicated time point, 10 μl of cell counting kit (CCK)8 solution (Dojindo, Kumamoto, Japan) was added to each well and incubated at 37° C for 2 h. Absorbance was then measured by an RT-6000 microplate reader (Rayto, Shenzhen, China) at a wavelength of 450 nm.

### Fluorescence *in situ* hybridization

HCT116 cells were immobilized with 4% paraformaldehyde for 20 min and then washed three times with phosphate-buffered saline (PBS) for 5 min each time. After 8 min of protease K treatment, cells were dehydrated using an ethanol gradient and then hybridized with a fluorescently labeled ABHD11-AS11 probe. 4',6-diamidino-2-phenylindole (Life Technologies, Carlsbad, CA, USA) was used to stain the nucleus. Fluorescence was observed under a Nikon microscope.

### Flow cytometric analysis of apoptosis

Cells were harvested and fixed with cold ethanol overnight and then incubated with propidium iodide and RNase in the dark for 15 min. They were washed twice with cold PBS and harvested, then stained with fluorescein isothiocyanate-conjugated annexin V for 20 min and propidium iodide for 15 min in the dark. Stained cells were evaluated by flow cytometry using the FACSAriaIII sorter (BD Biosciences, San Jose, CA, USA) and analyzed by FlowJo vX.0.7 software.

### Cell migration and invasion assays

Cell migratory and invasive abilities were detected with the Transwell assay kit (BD Biosciences). A total of 1 × 10^5^ cells were suspended in 200 μl serum-free medium and seeded in the upper compartment of the Transwell insert. Then, 500 μl of growth medium was added to the lower chambers as a chemoattractant. After 24 h, cells were fixed with 4% paraformaldehyde, and migratory cells on the underside were stained with 0.1% crystal violet. Cells in five random fields of view were counted under the DC 300F phase-contrast microscope (Leica, Wetzlar, Germany).

### Mouse xenograft study

Six-week-old male BALB/c nude mice were bought from the Experimental Animal Center of Xiamen University (Xiamen, China). HCT116-shNC and HCT116-shADHB11 cells were harvested and resuspended in PBS at 5 × 10^7^ cells/ml, and 100 μl of the cells was injected subcutaneously into the right flank of each mouse. The tumor size was recorded once a week. Tumor volume (V) was calculated using the formula: π/6 × length × width^2^.

### Western blot analysis

RIPA lysis buffer (Sigma-Aldrich, St Louis, MO, USA) containing protease inhibitors (Sigma) was used to extract total protein. Protein concentrations were determined using the bicinchoninic acid method (Pierce Chemical Co., Rockford, IL, USA). A total of 40 μg of total protein was resolved by 8% tris–glycine sodium dodecyl sulfate polyacrylamide gels and transferred to nitrocellulose membranes. After being blocked in blocking buffer containing 5% non-fat milk for 2 h at room temperature, the membranes were incubated overnight with primary antibodies at 4° C. After washing three times, the membranes were incubated with the horseradish peroxidase-conjugated secondary antibody (diluted 1:5000). The membranes were washed again and proteins were visualized using ECL Substrates (Millipore, Bedford, MA, USA). GAPDH was used as an internal control. The primary antibodies used in this study were: ITGA5, phosphorylated (p)-Fak, Fak, p-P85, P85, p-Akt1, and Akt1 (all Cell Signaling Technology, Beverly, MA, USA), and GAPDH (Santa Cruz Biotechnology, Santa Cruz, CA, USA).

### Statistical analysis

All statistical analyses were performed using GraphPad Prism version 8.0 (GraphPad Software, La Jolla, CA, USA). The Student’s t-test was used to estimate statistical significance between groups. A P-value of less than 0.05 was regarded as statistically significant. Results are shown as means ±standard deviation (SD) of three biological replicates or samples.

### Ethical statement

This study was approved by the Medical Ethics Committee of Affiliated Sanming Hospital of Fujian Medical University.

### Data availability statement

The data that support the findings of this study are available from the corresponding author upon reasonable request.

## RESULTS

### ABHD11-AS1 was overexpressed in CRC tissues and indicated poor prognosis

**Figure 1:**
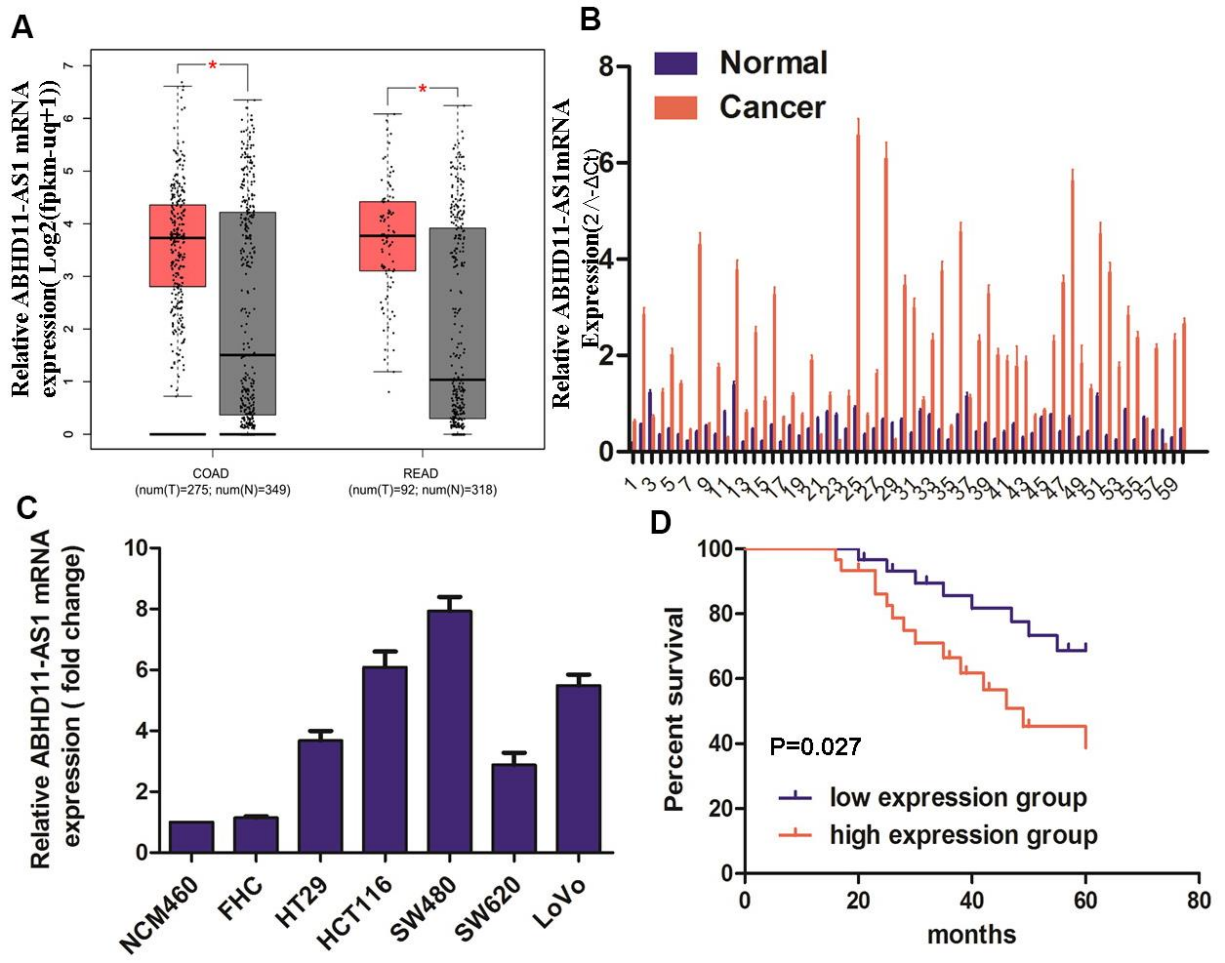
**ABHD11-AS1 was highly expressed in CRC and indicated a poor prognosis.** (**A**) Public database GEPIA displayed the mRNA expression of ABHD11-AS1 in CRC and normal colorectal tissue. (**B**) mRNA expression of ABHD11-AS1 in CRC and normal colorectal tissue in our patient samples. (**C**) mRNA expression ABHD11-AS1 in CRC and normal colonic epithelium cell line. (**D**) Kaplan–Meier plot of 60 patients with survival data (from our samples) stratified by ABHD11-AS1 expression levels.

Using Gene Expression Profiling Interactive Analysis (GEPIA), we investigated ABHD11-AS1 expression in CRC. As shown in Figure 1A, ABHD11-AS1 was overexpressed in CRC tumor samples compared with normal tissue samples. Quantitative reverse transcription (RT)-qPCR analysis showed that the expression of ABHD11-AS1 mRNA was increased in 86.7% (52/60) of CRC patients (Figure 1B). We also used RT-qPCR to investigate ABHD11-AS1 expression in five CRC cell lines and two normal human colonic epithelial cell lines. As shown in Figure 1C, ABHD11-AS1 expression was higher in HT29, HCT116, SW480, SW620, and LoVo colorectal cancer cell lines than in normal colonic epithelial cell lines NCM460 and FHC, with HCT116 and SW480 cells expressing much higher ABHD11-AS1 levels. Both cell lines were therefore used for subsequent studies.

CRC patients were divided into two groups according to their median expression of ABHD11-AS1, and Kaplan–Meier analysis indicated that the up-regulation of ABHD11-AS1 was associated with significantly shorter overall survival (Figure 1D, P = 0.027).

### ABHD11-AS1 deficiency attenuated proliferation and promoted apoptosis in CRC cells

**Figure 2:**
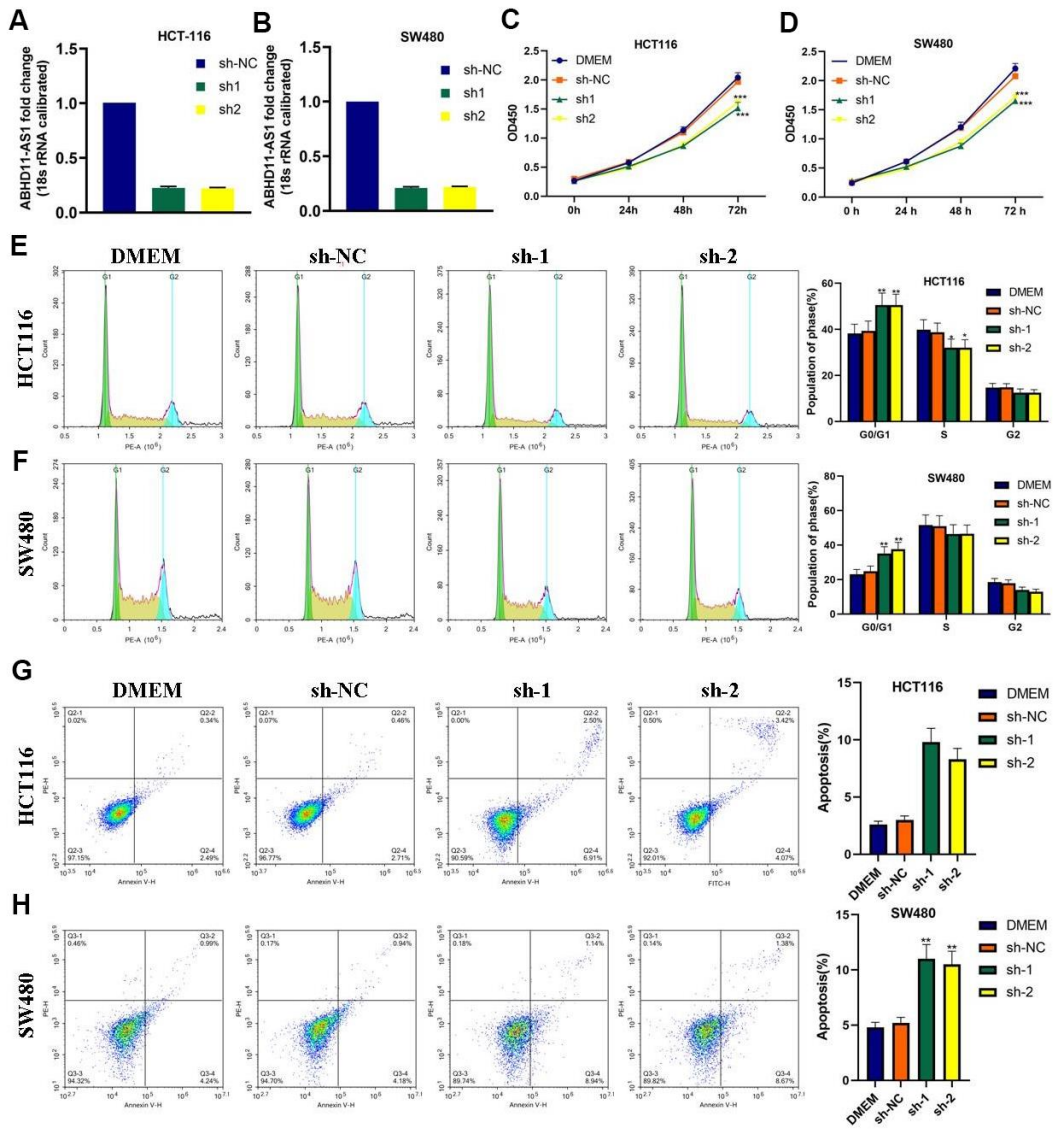
**ABHD11-AS1 deficiency attenuated proliferation and promoted apoptosis in CRC cells.** (**A**) ABHD11-AS1 expression in HCT116-ABHD11-AS1-sh cells was determined by RT-qPCR. (**B**) ABHD11-AS1 expression in SW480-ABHD11-AS1-sh cells was determined by RT-qPCR. (**C**) The proliferation curves of HCT116-sh-NC cells and HCT116-ABHD11-AS1-sh cells measured by CCK-8 array. (**D**) The proliferation curves of SW480-sh-NC cells and SW480-ABHD11-AS1-sh cells measured by CCK-8 array. (**E**) PI staining illustrated the cell cycle distribution of HCT116-sh-NC cells and HCT116-ABHD11-AS1-sh cells. (**F**) PI staining illustrated the cell cycle distribution of SW480-sh-NC cells and SW480-ABHD11-AS1-sh cells. (**G**) Annexin V-7ADD and PI double staining illustrated the apoptosis cells of HCT116-sh-NC cells and HCT116-ABHD11-AS1-sh cells. (**H**) Annexin V-7ADD and PI double staining illustrated the apoptosis cells of SW480-sh-NC cells and SW480-ABHD11-AS1-sh cells. *P<0.05, **P<0.01, ***P<0.001.

Next, we used lentiviruses to infect HCT116 and SW480 cell lines with ABHD11-AS1 shRNAs to knock-out ABHD11-AS1 expression. As demonstrated in Figure 2A, 2B, ABHD11-AS1 expression was obviously decreased following ABHD11-AS1-sh-1 or ABHD11-AS1-sh-2 infection. CCK-8 assay findings indicated that the ABHD11-AS1 deficiency significantly impaired proliferation in HCT116 and SW480 cells (Figure 2C, 2D), while fluorescence-activated cell sorting results revealed that more ABHD11-AS1-deficient cells were arrested in G0/G1 phase compared to the control group (Figure 2E, 2F). Moreover, as seen in Figure 2G, 2H, sh-ABHD11-AS1 infection significantly increased the apoptosis rate in CRC cells. These results suggest that ABHD11-AS1 promotes growth and inhibits apoptosis in CRC cells.

### ABHD11-AS1 deficiency suppressed CRC cell migration and invasion

**Figure 3:**
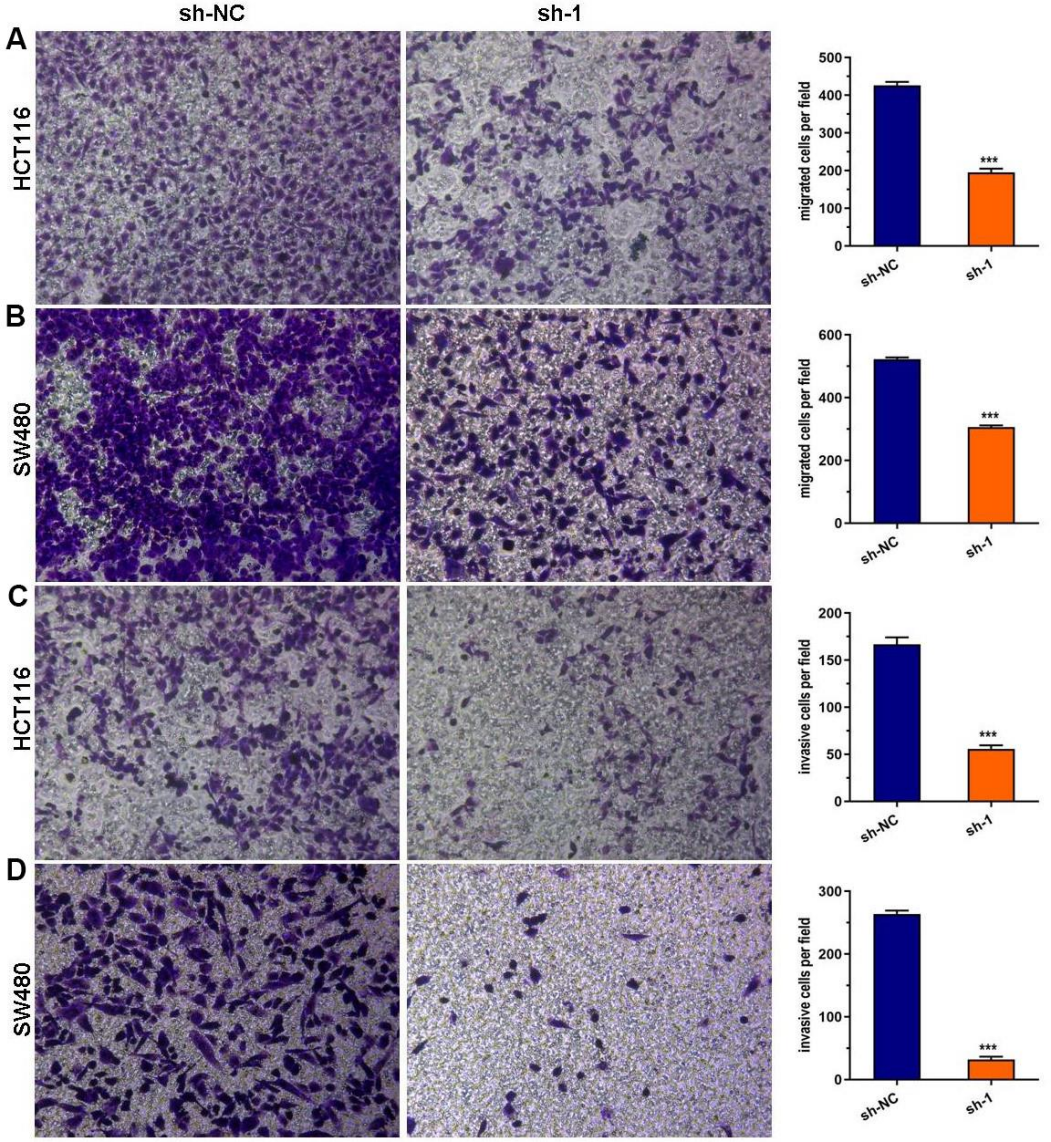
**ABHD11-AS1 deficiency suppressed CRC cell migration and invasion.** (**A**) Transwell assay for cell migration. The ABHD11-AS1-deficiency HCT116 cell had significantly less migration cells than that of control. (**B**) Transwell assay for cell migration. The ABHD11-AS1-deficiency SW480 cell had significantly less migration cells than that of control. (**C**) Transwell assay for cell invasion. The ABHD11-AS1-deficiency HCT116 cell had significantly less invasion cells than that of control. (**D**) Transwell assay for cell invasion. The ABHD11-AS1-deficiency SW480 cell had significantly less invasion cells than that of control. ***P<0.001.

Using the Transwell assay, we discovered that a deficiency of ABHD11-AS1 dramatically attenuated the migration and invasion of HCT116 and SW480 cells (Figure 3A–3D).

### ABHD11-AS1 knockdown inhibited xenograft tumor growth *in vivo*

**Figure 4:**
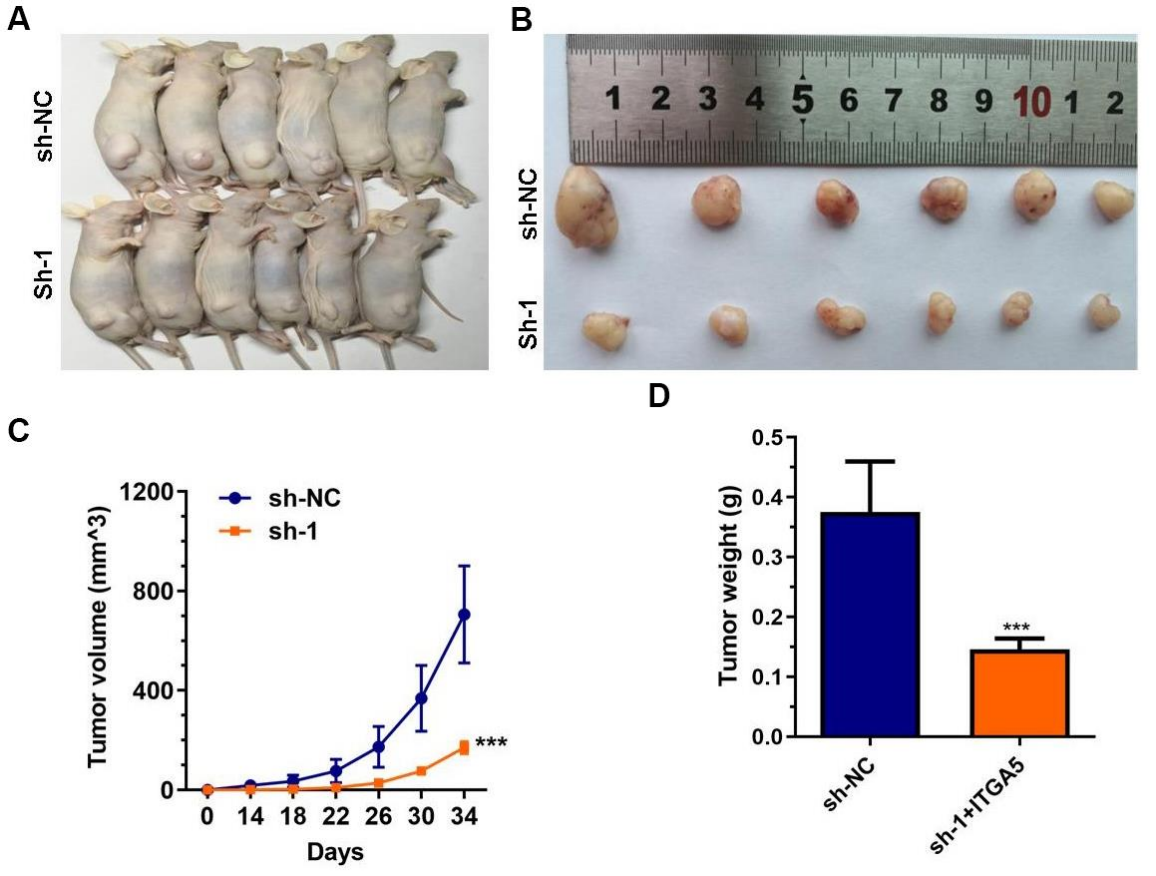
**ABHD11-AS1 deficiency impaired CRC cells growth *in vivo*.** (**A**) Gross view of the tumor bearing in the mice. (**B**) Gross view of after sampling. (**C**) Growth curve of tumor volume. (**D**) Tumor weight was significantly different between the control and ABHD11-AS1 deficiency groups. ***P<0.001.

To explore the oncogenesis of ABHD11-AS1 *in vivo*, athymic nude mice were subcutaneously injected with control cells or ABHD11-AS1-deficient cells. Tumor growth was then measured over the next 4 weeks. As shown in Figure 4A–4D, tumor volume and weight were significantly impaired in mice injected with ABHD11-AS1-deficient cells. These findings provide further support of an oncogenic role for ABHD11-AS1.

### ABHD11-AS1 deficiency inhibited the ITGA5/Fak/PI3K/Akt signaling pathway in CRC

**Figure 5:**
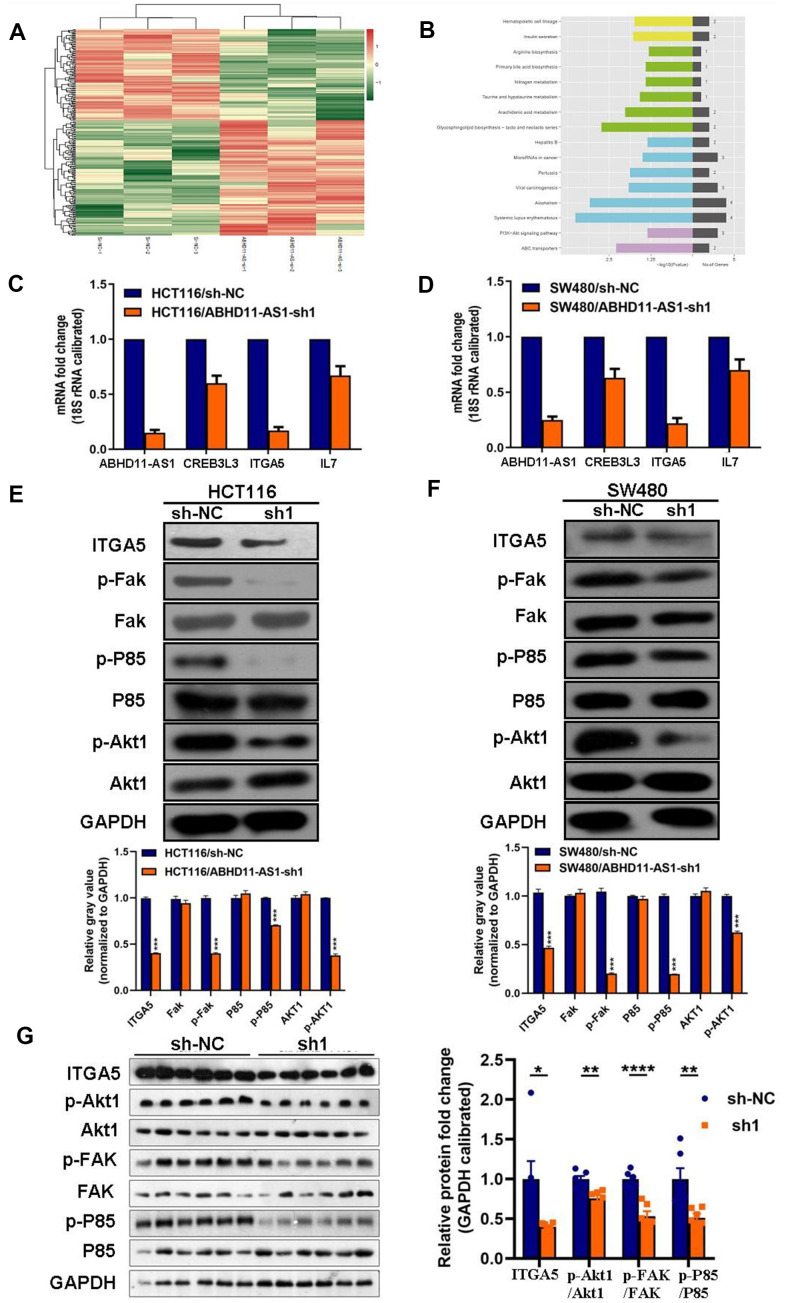
**ABHD11-AS1 deficiency inhibited ITGA5/Fak/PI3K/Akt signaling pathway in CRC.** (**A**) mRNA sequencing to find differential genes between HCT116-sh-NC cells and ABHD11-AS1 deficient cells and displayed via Heat-map. (**B**) Differential genes enriched by KEGG pathway. Top 16 pathways were illustrated. (**C**) RT-qPCR to detect mRNA expression of ITGA5, CREB3L3, ITGA5, IL7 in HCT116 cells and ABHD11-AS1 deficient cells. (**D**) RT-qPCR to detect mRNA expression of ITGA5, CREB3L3, ITGA5, IL7 in SW480 cells and SW480 ABHD11-AS1 deficient cells. (**E**) WB analysis of protein abundance of ITGA5, FAK, p-FAK, P85, p-P85, Akt1, p-Akt1 in HCT116 cells and HCT116 ABHD11-AS1 deficient cells. (**F**) WB analysis of protein abundance of ITGA5, FAK, p-FAK, P85, p-P85, Akt1, p-Akt1 in SW480 cells and SW480 ABHD11-AS1 deficient cells. (**G**) WB analysis of protein abundance of ITGA5, FAK, p-FAK, P85, p-P85, Akt1, p-Akt1 in tumor tissues of nude mice. ***P<0.001.

We next employed transcriptome sequencing to investigate the differential expression of genes after the down-regulation of ABHD11-AS1 in CRC cells (Figure 5A). Kyoto Encyclopedia of Genes and Genomes (KEGG) enrichment analysis demonstrated that PI3K/Akt signaling pathway-related genes such as cAMP responsive element-binding protein 3-like 3 (*CREB3L3*), integrin-α5 (*IGT5A*), and interleukin-17 (*IL-17*) were down-regulated in shABHD11-AS1 over-expressing cells (Figure 5B). We verified these results using RT-qPCR and found that *IGT5A* mRNA expression decreased dramatically in ABHD11-AS1 knockdown cells (Figure 5C, 5D). shABHD11-AS1 regulation of *ITGA5* mRNA expression was further confirmed using small interfering RNA transfection (data not shown).

**Figure 6:**
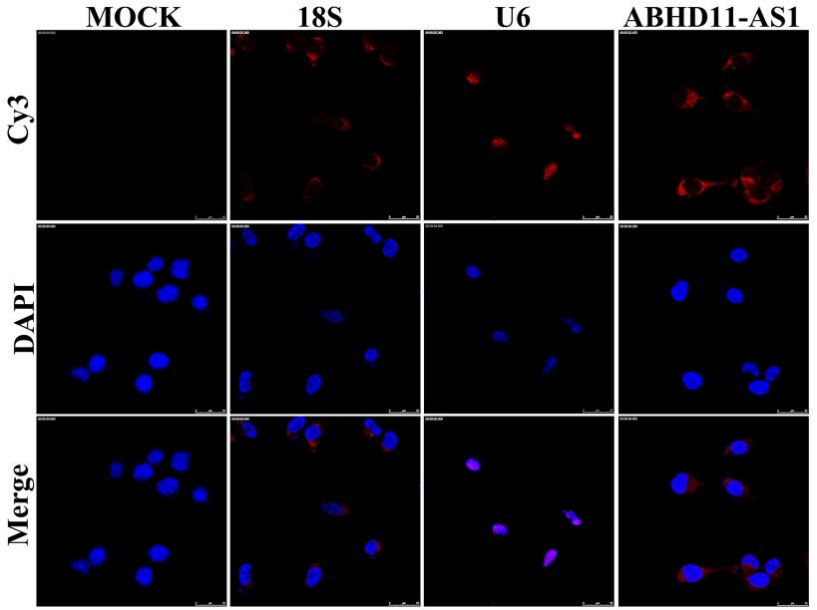
**ABHD11-AS1 was mainly localized in the cytoplasm.** Subcellular localization of ABHD11-AS1 determined by RNA FISH assay, U6 and 18s rRNA were used as internal controls.

Next, we analyzed the protein expression of ITGA5 and other proteins downstream of ITGA5 in ABHD11-AS1 knockdown cells. We showed that the expression of ITGA5, p-FAK, p-P85, and p-Akt1 was dramatically decreased following sh-ABHD11-AS1 infection in two CRC cell lines (Figure 5E, 5F). Similar results were observed in the tumor tissues of nude mice (Figure 5G). To explore the underlying mechanism whereby ABHD11-AS1 regulates ITGA5 expression, we determined the subcellular localization of ABHD11-AS1 in CRC cells. RNA fluorescence *in situ* hybridization indicated that ABHD11-AS1 was mainly localized in the cytoplasm (Figure 6). Studies have shown that lncRNAs localized in the cytoplasm regulate mRNA turnover by competitively binding micro (mi)RNAs, but unfortunately we failed to identify miRNAs that could bind to both ABHD11-AS1 and ITGA5 mRNA simultaneously (data not shown).

### ITGA5 abrogated the effects of ABHD11-AS1 deficiency in CRC

**Figure 7:**
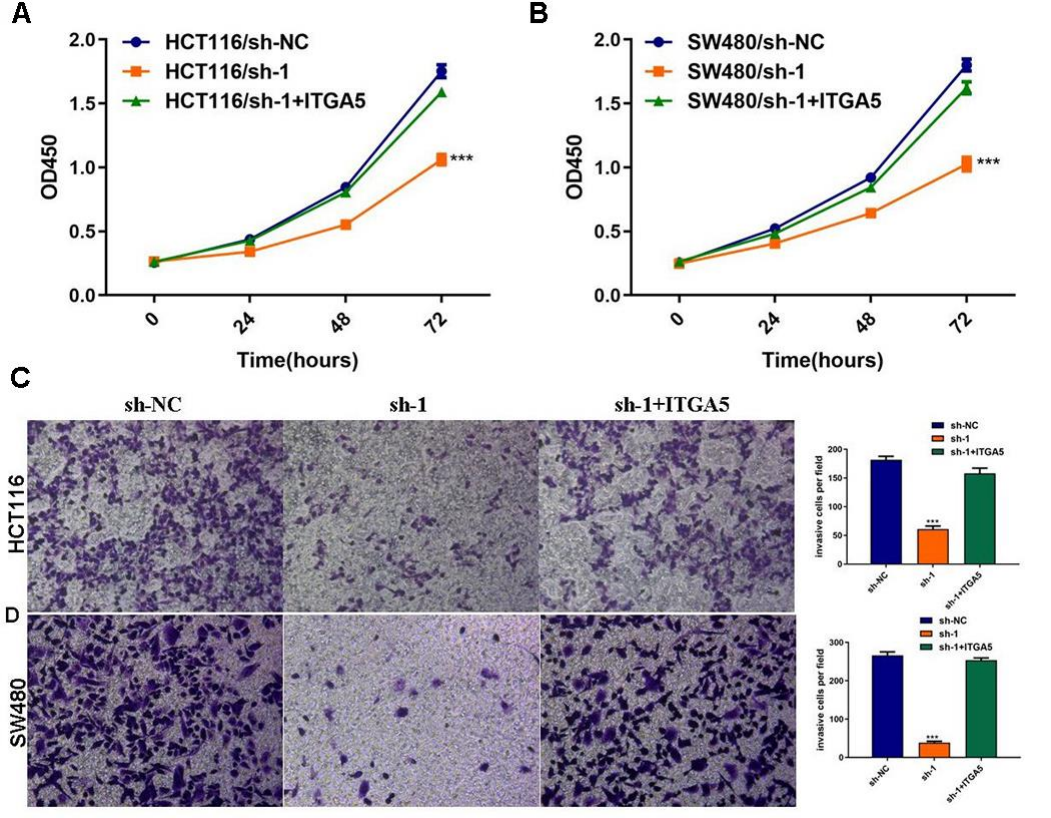
**ITGA5 was able to rescue the effects of ABHD11-AS1 deficiency in CRC.** (**A**) The proliferation curve of HCT116 cells with indicated administrations measured by CCK-8 assay. (**B**) The proliferation curve of SW480 cells with indicated administrations measured by CCK-8 assay. (**C**) Transwell assay to analysis invasion of HCT116 cell with indicated administrations. (**D**) Transwell assay to analysis invasion of SW480 cell with indicated administrations. ***P<0.001.

To explore whether sh-ABHD11-AS1 exerted its function through regulating the ITGA5/Fak/PI3K/Akt signaling pathway in CRC, we transfected HCT116 and SW480 cells with sh-ABHD11-AS1 lentiviruses alone or in combination with ITGA5 lentiviruses. As shown in Figure 7A, 7B, ITGA5 over-expression abrogated the ABHD11-AS1 inhibition-mediated effect on CRC cell proliferation. ITGA5 was also able to rescue the cell invasive abilities inhibited by sh-ABHD11-AS1 (Figure 7C, 7D).

## DISCUSSION

Mutations accumulating in chromatin can initiate and boost tumor development and progression, while epigenetic alterations themselves can be attributed to carcinogenesis. Additionally, lncRNA dysfunction has been reported to be associated with the development of a variety of different types of cancer [15–17].

LncRNAs can be oncogenes or tumor suppressor genes that function to regulate cellular differentiation and proliferation [18], and mounting evidence suggests that they have the potential to be used as precise non-invasive biomarkers for CRC screening and disease evaluation [19]. For instance, H19, an oncogenic lncRNA in CRC, was shown to participate in cell proliferation and migration, and to be a predictor of poor prognosis [9].

The expression of ABHD11-AS1 is well studied in human papillary thyroid cancer [20], pancreatic cancer [21], and bladder cancer [22]. Herein, we observed that ABHD11-AS1 was up-regulated in CRC tissues, and that its high expression predicted unfavorable clinical outcomes. Additionally, ABHD11-AS1 depletion suppressed the proliferation and invasiveness of CRC cells *in vitro* and *in vivo*, suggesting that ABHD11-AS1 exerts an oncogenic effect in CRC.

Mechanisms by which lncRNAs regulate gene expression are dependent on its subcellular localization. Studies have shown that lncRNAs localized in the cytoplasm can function as miRNA sponges to inhibit binding to mRNA targets, leading to the stabilization of target mRNAs and regulation of corresponding protein expression [23]. The cytoplasmic localization of ABHD11-AS1 in CRC cells observed in the present study implies that it may function as a competitor of an miRNA that targets *ITGA* mRNA; however, this is yet to be identified.

The PI3K/Akt signaling pathway is an important pathway that regulates cell metabolism, proliferation, and survival, and is frequently constitutively activated in cancer. This can be via activating mutations of p110a (*PIK3CA*), fibroblast growth factor receptor 3, Ras, and Akt kinase genes, or inactivating mutations of the negative regulator phosphatase and tensin homolog gene [24]. During the past decade, many lncRNAs have been shown to contribute to cancer development, metastasis, and drug resistance through activating the Akt pathway. For example, ABHD11-AS1 was found to increase levels of p-PI3K and p-Akt1 proteins in pancreatic cancer cells, although the detailed upstream mechanisms remain unknown [13]. In this study, transcriptome sequencing and KEGG enrichment analysis showed that the PI3K/Akt signaling pathway may contribute to the tumor-promoting function of ABHD11-AS1.

Integrins are heterodimeric transmembrane receptors consisting of α and β subunits, which form a large family that participates in cell proliferation, cytoskeletal organization, adhesion, migration, and differentiation [25, 26]. ITGA5 and FAK are two important upstream effectors of the PI3K/Akt pathway [27, 28]. The cytoplasmic tyrosine kinase FAK has been identified as a key mediator of integrin intracellular signaling. FAK phosphorylation acts like a switch of downstream signaling cascades, and is essential for the activation of PI3K/Akt signaling [29]. ITGA5 dysregulation was shown to facilitate the occurrence and development of CRC, but its precise role in CRC remained controversial [30–32]. Here, we showed that sh-ABHD11-AS1 decreased the mRNA and protein expression of ITGA5 in CRC cell lines, resulting in the down-regulation of p-FAK, p-P85, and p-Akt1. Furthermore, we found that ITGA5 over-expression abrogated the inhibitory effects of ABHD11-AS1 deficiency on CRC cell proliferation, migration, and invasion. These results suggest that ABHD11-AS1 exerts its tumor-promoting function through targeting the ITGA5/Fak/PI3K/Akt signaling pathway.

Integrins have been identified as therapeutic targets for cancer metastasis, with several integrin-based antibodies investigated in clinical trials [33]. As an example, the ITGA5 antagonist cilengitide has anti-cancer effects on metastatic tumors in animal models [34]. Additionally, the ITGA5-based antibody CTNO-95 promoted progression-free survival in patients with castration-resistant prostate cancer [35]. Our results suggest another possible means of modulating ITGA5 through orchestrating ABHD11-AS1 expression. ABHD11-AS1 could also be used as a prognostic predictor and is a promising target for CRC therapy.
